# The Neuroprotective Properties of Shogaol-Enriched Ginger Extract (SEGE) Mitigate Synaptic, Cholinergic, and Metabolic Brain Dysfunction in a Mouse Model of Metals and High-Fat-Diet-Induced Neuropathology

**DOI:** 10.3390/biomedicines14102208

**Published:** 2026-09-29

**Authors:** Sara Ishaq, Armeen Hameed, Amna Liaqat, Rabia Basri, Sohana Siyar, Syed Ghulam Musharraf, Amna Jabbar Siddiqui, Zaman Ashraf, Sher Qadar, Touqeer Ahmed

**Affiliations:** 1 Neurobiology Laboratory, Department of Biomedicine, Atta-ur-Rahman School of Applied Biosciences, National University of Sciences and Technology, Sector H-12, Islamabad 44000, Pakistan; 2 Planning Commission, Ministry of Planning, Development, & Special Initiatives (MoPD&SI), Government of Pakistan, Pak Secretariat, Islamabad 44000, Pakistan; 3 H.E.J. Research Institute of Chemistry, International Center for Chemical and Biological Sciences, University of Karachi, Karachi 75270, Pakistan; 4 Dr. Panjwani Center for Molecular Medicine and Drug Research, International Center for Chemical and Biological Sciences, University of Karachi, Karachi 75270, Pakistan; 5 Department of Chemistry, Faculty of Sciences, Rawalpindi Women University, Rawalpindi 46300, Pakistan; 6 Department of Chemistry, Allama Iqbal Open University, Islamabad 44000, Pakistan

**Keywords:** neuropathology, neurotoxic metals, high-fat diet, Shogaol, synaptic plasticity

## Abstract

**Background/Objective:** Co-exposure to heavy metals from environment and a high-fat diet (HFD) represents a growing health concern, contributing to various diseases, but their combined effects on brain health remain less explored. This study aimed to evaluate the neuroprotective potential of Shogaol-enriched ginger extract (SEGE) against heavy metals- and HFD-induced neuropathology. Plant-derived isolated pure compounds can be costly and less accessible; SEGE may offer a translatable, multi-target dietary intervention. **Methods:** Male Balb/c mice (8–11 weeks old) were exposed to a metal mixture of arsenic (As), lead (Pb), and aluminum (Al; 25 mg/kg/day each) in drinking water and 40% HFD in feed for 60 days. SEGE was administered via feed at two different doses (2 mg/kg/day and 12 mg/kg/day). Assessments including gene expression analyses (using Quantitative Reverse Transcription Real Time Polymerase Chain Reaction (qRT-PCR)) of synaptic and cholinergic markers, spectrophotometric measurement of acetylcholine (ACh) levels, neuronal counting in the cortex and hippocampus via histology, and Gas chromatography mass spectrometric (GC/MS) analysis for brain metabolic quantification were performed. **Results:** The combined toxic exposure significantly downregulated the expression of synaptic plasticity markers (Synaptophysin, Polysynaptic Density Protein 95 (*PSD95*), and Calcium/Calmodulin-Dependent Protein Kinase-IV (*CAMK-4*)) and α and β Nicotinic Acetylcholine Receptors (*α7nAChR*, *α4nAChR*, and *β2nAChR*) in the hippocampus and cortex. ACh levels were also reduced along with significant neuronal loss in the cortical layers and the hippocampal regions. Met + HFD disrupted the brain’s metabolic profile. SEGE treatment significantly restored these markers and attenuated the cholinergic deficits. SEGE treatment, specifically at a higher dose, demonstrated a rescuing effect on the brain’s metabolic profile when compared to the metals and HFD. SEGE further preserved the neuronal count at a higher dose (12 mg/kg/day), exhibiting superior protective effects. **Conclusions:** These findings suggest that SEGE mitigates neurotoxicity induced by the interaction of toxic metals and dietary factors, likely by preserving synaptic plasticity, metabolic profile, and cholinergic functions, particularly at a higher dose.

## 1. Introduction

The modern world is currently facing two serious threats, i.e., growing industrialization and rapid urbanization [1]. Increased global industrialization is the primary source of the release of toxic heavy metals such as arsenic (As), mercury (Hg), cadmium (Cd), lead (Pb), and chromium (Cr) into the environment, leading to elevated human exposure to these metals [2,3,4]. Despite the World Health Organization (WHO) and Pakistan’s Environmental Protection Agency (EPA) having set the minimum permissible levels of heavy metals to <0.01–0.05 mg/L, the detected levels of these metals in food and drinking water are far higher (101–501 mg/L in the case of As) in Pakistan [5]. Other than the heavy metals, aluminum (Al; permissible level < 0.02 mg/L) is another metal that, although naturally abundant in Earth’s crust, has recently increased in food and water resources due to its increased use in the food industry [6]. These toxic metals can enter the human body through inhalation, food and water consumption, and, at times, direct skin absorption, resulting in accumulation in vital organs and causing major health dysfunction [7].

Many studies involving different metals, such as As, Pb, Cd, Hg, and Al, either individually or in combination, have demonstrated their toxic effects, particularly on the nervous system [8,9]. As is the highest detected metal in drinking water and is known to accumulate in the liver, kidneys, and brain [10]. As is usually present in trivalent (As^3+^) or pentavalent (As^5+^) forms, which are hypothesized to replace the phosphate group of adenosine triphosphate (ATP), thus blocking energy production in the cells [11]. In the brain, it causes disrupted synaptic plasticity, neurodegeneration, and damage to deoxyribonucleic acid (DNA) and proteins by increasing oxidative stress [12]. Pb is another toxicant that affects the brain in a similar manner, i.e., by increasing reactive oxygen species (ROS) production, deposition of amyloid beta (Aβ) plaques, disrupting cholinergic projections, causing apoptotic neuronal death, damaging the blood–brain barrier (BBB), and disrupting synaptic plasticity [13,14]. This ultimately results in learning and memory issues, especially in children [15]. While Al is not a heavy metal, it is bioavailable even at low concentration which deems it a neurotoxicant [16]. The exact mechanism of Al neurotoxicity is yet to be known, but the metal is thought to cross the BBB by replacing iron from iron transport protein [17] and initiates the process of neurodegeneration [18]. While the individual neurotoxic mechanisms of these metals are well-established, humans are rarely exposed to a single toxicant at a time. Studies have consistently demonstrated that humans are frequently exposed to a combination of metals and that the combination of metals is more toxic than individual metals. One such study on a combination of As, Pb, and manganese (Mn) showed highest accumulation of only Pb in the brain, which could be attributed to the presence of Pb-binding proteins in the brain [19]. Another study using a combination of metals (As, Pb and Cd) reported an increased neuronal deterioration in rat pups, which was higher than the individual effects of these metals [20].

It is important to mention that a critical and less well explored variable in this toxicological equation is the lifestyle. The global rise in the consumption of western diet or the HFD, which was reported to be 9.4% in 2010 [21], parallels the rise in environmental pollution [21,22]. Pakistan is recognized as the second-highest consumer of fatty acids globally, a trend that has placed additional strain on its gross domestic production (GDP) [23]. Increased consumption of HFD impairs brain function, cognition [24], and learning by reducing brain-derived neurotropic factor (*BDNF*) [25], increasing ROS, causing mitochondrial dysfunction, disrupting synaptic plasticity, and inducing neurodegeneration and neurovascular decline [26,27]. This emphasizes the need to assess the effects of toxic metals combined with HFD on mental health. One study has reported the neurotoxic effects of high-cholesterol diet in combination with copper (Cu) [28]. We have also previously shown that a combination of Al, Cd, Pb, and Cu with HFD caused an increased aging-like neurodegeneration and cognitive decline in mice [22], but more supporting evidence is required so that treatments can be devised accordingly.

Natural compounds are increasingly employed for treating health issues due to their cost-effectiveness, low side effects, availability, and substantial therapeutic potential [29]. Ginger (*Zingiber officinale*) contains potent phenols, primarily 6-Gingerol (GN) and 6-Shogaol (SG). Notably, 6-SG, the hydroxy-derivative of 6-GN produced during thermal processing, exhibits better anti-inflammatory, anti-diabetic, anti-tumor, anti-oxidative, neuroprotective, and anti-amyloidogenic effects compared to its precursor [30,31,32,33]. Given the fact that the pure compound isolation (either SG or GN) can be costly for a larger use, use of SEGE, obtained via thermal conversion, may provide a translatable and accessible therapeutic strategy.

Consequently, the present study was designed to closely mimic the real-life scenarios where humans are exposed simultaneously to multiple toxic metals from industrial and geographical leaching [2,3,4], along with the increased trend of HFD consumption in society [21]. As SEGE is a bioactive compound, it is also consumed in routine diet on a daily basis; therefore, evaluating the therapeutic potential of this plant extract in metals and HFD-induced neuropathology was the prime target. The study evaluated the ameliorative potential of chemically characterized SEGE on two critical neurofunctional pathways, i.e., synaptic plasticity (assessed via Synaptophysin, *PSD95*, and *CAMK-4* markers) and cholinergic system (assessed via ACh levels and *α7nAChR*, *α4nAChR*, and *β2nAChR* receptors expression). This study provides a comprehensive insight into the combined neurotoxicity of environmental and dietary factors and the efficacy of Shogaol-enriched intervention.

## 2. Materials and Methods

### 2.1. Animal Groups

A total of 40 male *Balb/c* mice (8–11 weeks old) were obtained from Laboratory Animal House, Atta ur Rahman School of Applied Biosciences (ASAB), National University of Sciences and Technology (NUST), Islamabad, and were kept in 8 plastic cages (40 cm × 25 cm × 15 cm), with 5 mice in each cage. Standard housing conditions were maintained on 14/10 light–dark cycle (representing seasonal variations observed in natural environment), 22 ± 2 °C temperature, and feed and water ad libitum. The Institutional Review Board (IRB) of ASAB, NUST, Pakistan, reviewed and approved the study (IRB #135). All the experiments were conducted in accordance with the guidelines of the Institute of Animal Laboratory Research (IALR), Division on Earth and Life Sciences (DELS), National Institute of Health (NIH), USA [34].

### 2.2. Chemicals

Aluminum chloride hexahydrate (AlCl_3_·6H_2_O), lead acetate trihydrate Pb (CH_3_COO)_2_·3H_2_O, and sodium acetate (CH_3_COONa) were purchased from Scharlau, Barcelona, Spain, Sigma-Aldrich, München, Germany, and Sigma-Aldrich, Saint Louis, MO, USA, respectively. The rest of the chemicals were also purchased from Sigma-Aldrich, Saint Louis, MO, USA, which include sodium arsenate (Na_3_AsO_4_), hydrochloric acid (HCl), sodium hydroxide (NaOH), hydroxylamine hydrochloride (NH_2_OH·HCl), ferric chloride hexahydrate (FeCl_3_·6H_2_O), acetylcholine chloride (C_7_H_16_ClNO_2_), and absolute ethanol (C_2_H_6_O). Taq polymerase, reverse transcriptase, deoxyribonucleotide triphosphate, and SYBR Green were obtained from Thermo Scientific, Waltham MA, USA. Trizole was purchased from Ambion/RNA by Life technologies, Waltham MA, USA.

### 2.3. Metals Mixture and HFD Preparation

Metals were given at the dose of 25 mg/kg/day each (75 mg/kg/day combined) for 2 months mixed in the drinking water of the animals. This dose of metals was selected in a way which would be non-lethal but would represent the natural metal-contaminated drinking water resources [4,35,36,37]. To ensure a stable and consumable water solution for the animals, the pH of the solution was maintained at 3.82 to ensure solubility. This pH was also quite similar to the pH (3–4) of natural metal-contaminated drinking water resources, carbonated drinks, and commercially available drinking waters that are also being regularly used by humans [38,39]. The pH of the control group’s drinking water remained between 6.5 and 7.0. The water intake was recorded for each group throughout the 2 months’ time, which was not more than 10 mL/animal/day and there was no significant difference between any groups. The metals were given via drinking water to avoid the high stress of daily intragastric gavage, which can induce systemic corticosterone responses and confound the study of neurodegeneration and anxiety.

HFD (40%) was prepared by adapting the already described method [22] after obtaining buffalo adipose tissue fat from a meat market and then melting it in the laboratory. Briefly, the buffalo fat was melted, filtered, and then mixed with the standard rodent chow in a 40:60 (*w*/*w*) ratio.

### 2.4. Preparation, Characterization, and Dosing of SEGE

**Figure 1:**
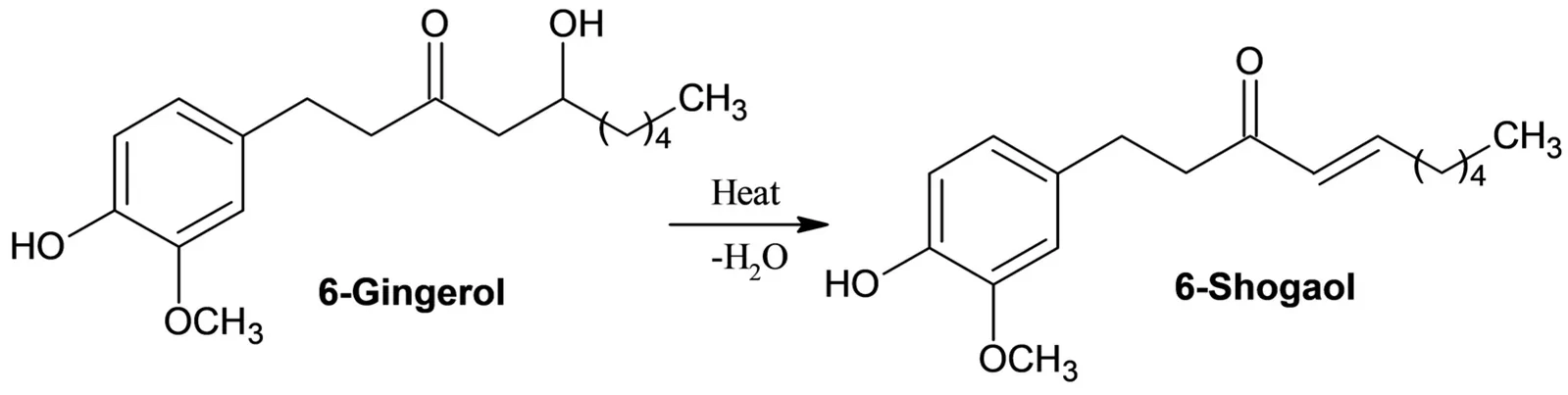
Conversion of 6-gingerol into 6-SG upon heating by dehydration.

Fresh ginger rhizomes were purchased from the local market and were washed, sliced, and dried at 80 °C in the laboratory. The dried material was pulverized into fine powder. The extraction was performed at an elevated temperature to maximize the conversion of 6-GN to 6-SG (Figure 1). Briefly, 25 g of dried ginger powder was mixed with 250 mL of ethanol (95%) in a Soxhlet apparatus at 80 °C for 4 h and left overnight. The mixture was filtered, centrifuged at 5000 rpm for 20 min at room temperature, and the supernatant was filtered through a 0.2 μm nylon membrane. Excess ethanol was removed via rotatory evaporation.

**Figure 2:**
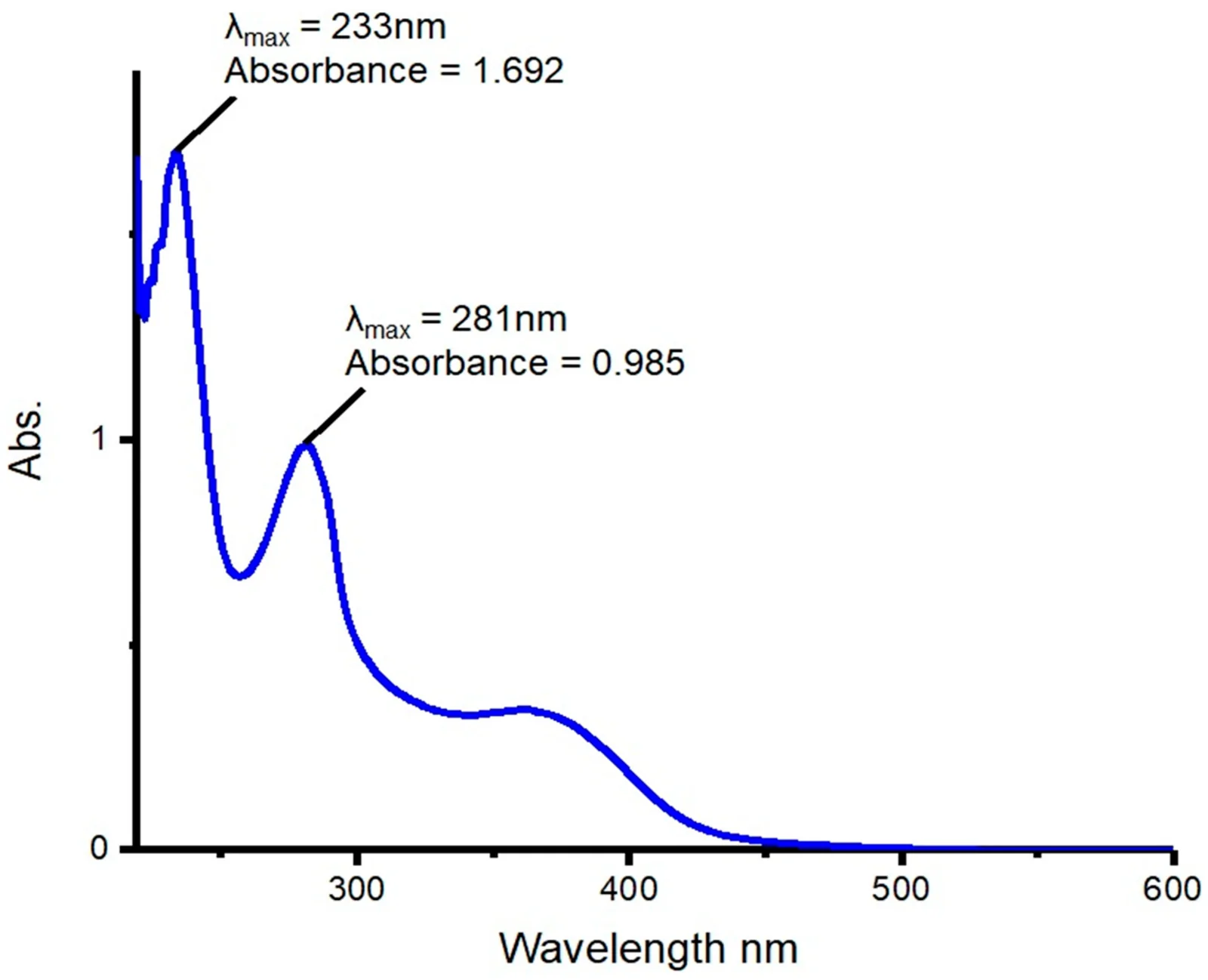
UV spectrum of the ethanolic extract of ginger showing two absorption maxima.

Characterization was performed via high-performance liquid chromatography (HPLC) using an acetonitrile water ingredient. UV–visible spectrophotometer (Shimadzu 1800 series, Shimadzu Corporation, Kyoto, Japan) and FTIR spectrophotometer (Perkin Elmer, Inc., Shelton, CT, USA) were used to confirm functional groups. The dried extraction method yielded an SEGE containing approximately 57–60 mg/g of 6-SG and 20–25 mg/g of 6-GN. The presence of α,β-unsaturated carbon moiety was confirmed via UV absorption at 281 nm and FTIR stretching at 1633 cm^−1^ (Figure 2).

This SEGE was given via dietary supplementation to avoid the stress associated with oral gavage. Based on the HPLC characterization (57–60 mg/g 6-SG extract), SEGE was added to the diet to deliver target doses (2 mg/kg/day as a low dose and 12 mg/kg/day as a high dose) of the total extract. The purpose of selecting two different treatment doses, i.e., a low dose (2 mg/kg/day) and a 6-times higher dose (12 mg/kg/day), was to provide a dose range demonstrating pharmacological effect in reasonable therapeutic range from already published SG doses [40,41,42,43,44,45,46,47,48,49]. A stock solution of SEGE was prepared at a concentration of 50 mg/mL in absolute ethanol. To prepare the experimental diets, calculated volumes of the stock solution were added thoroughly into the powdered HFD to achieve the required concentrations, based on the average daily feed consumption of the mice. The ethanol vehicle was allowed to evaporate completely prior to pelleting and feeding. The SEGE-added feed was prepared fresh weekly to ensure stability.

### 2.5. Experimental Design

**Figure 3:**
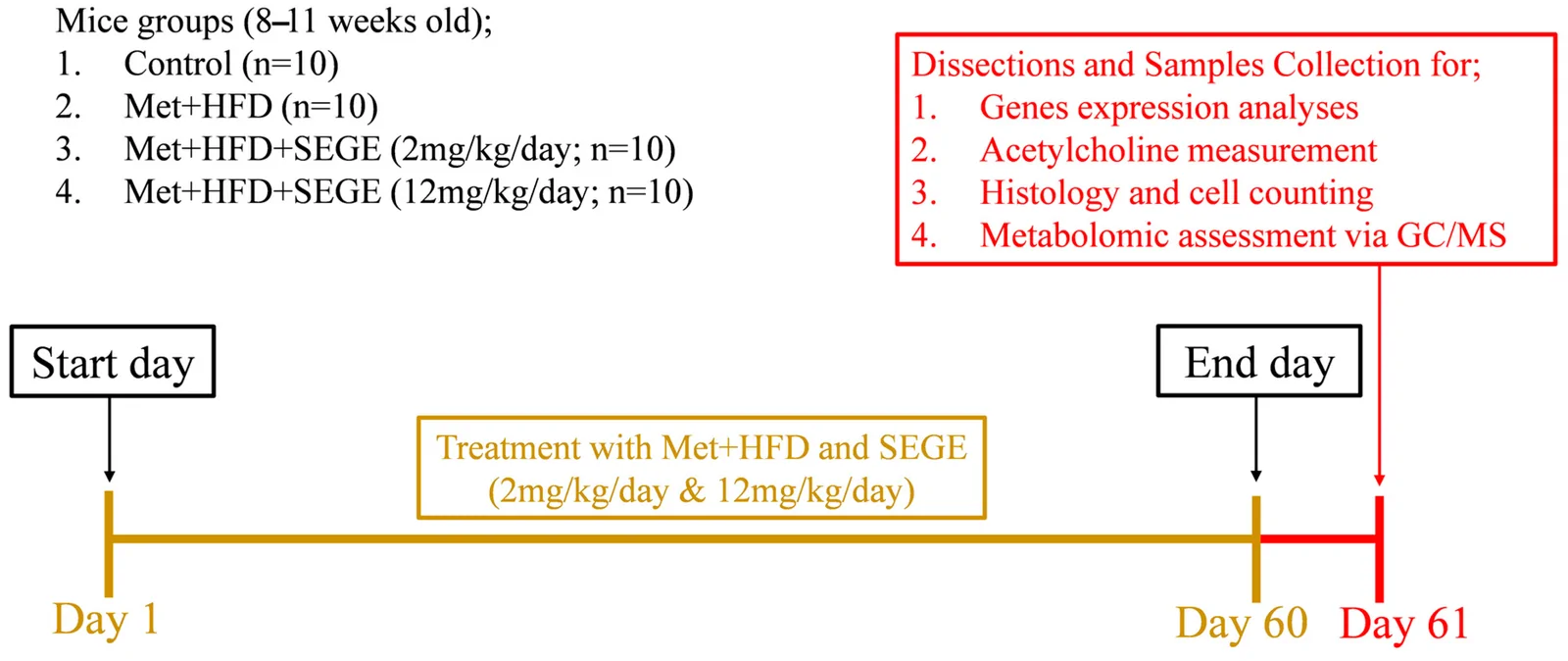
Study timeline.

The animals were randomly divided into 4 groups, i.e., control group (no treatment); Met + HFD (metals+ high-fat diet) group; Met + HFD + SEGE (2 mg/kg/day) treated group; Met + HFD + SEGE (12 mg/kg/day) treated group, with 10 animals in each group (Table 1). The study duration was 61 days. The animal model was developed with a 60-day treatment with metals, HFD, and SEGE. Animals were dissected on the 61st day (Figure 3). One subset of the animals (5 animals from each group) was dissected for brain samples for genes, ChAT, and ACh analysis, and GC/MS analysis where the other subset (remaining 5 animals in each group) was dissected via heart perfusion for histology and cell counting.

### 2.6. Gene Expression Analysis

Following a 60-day treatment, mice were sacrificed, and hippocampal and cortical tissues were obtained. Total RNA was extracted using a Trizol reagent [50]. cDNA synthesis was carried using Thermo Scientific, Walmath MA, USA, reverse transcriptase kit from normalized RNA concentrations as per the manufacturer’s protocol. RT-PCR was performed using SYBR Green. Relative gene expression was calculated using Livak method ∆∆CT [51] with *GAPDH* as the housekeeping gene. In accordance with the standard ∆∆CT (Livak) method for relative gene expression, the control group is defined as the calibrator sample and is normalized to a value of 1.00. As this value is the reference point for calculating the fold-change of all other experimental groups, the error (±0.0) is intrinsic to the method’s definition of the reference group. Primers’ sequences (efficiencies between 99–106%) are given in Table 2.

### 2.7. Biochemical Analysis for ACh Levels in the Brain

ACh levels were measured using a previously described method [52]. Tissues were boiled, homogenized, and centrifuged. The supernatant was reacted with 300 µL alkaline hydroxylamine (freshly prepared in NaOH in 1:1 by volume) and 100 µL of hydrochloric acid (HCL) solution (0.1 N solution in distilled water), followed by 100 µL ferric chloride solution (0.3 M solution in 0.1 N HCL). Absorbance of ACh was measured at optical density of 540 nm via BioRad^®^ PR 4100 absorbance reader by BioRab Laboratories, Inc., Hercules, CA, USA. Concentrations were determined against a standard curve of ACh chloride.

### 2.8. Neuronal Counting in Cortex and Hippocampus

A subset of mice (*n* = 5/group) underwent heart perfusion with saline followed by storage with 4% paraformaldehyde at 4 °C for 24 h [53]. Later, brains were paraffin embedded and sliced into 6 µm thickness (each) sections. These sections were stained with cresyl violet (Nissl staining) to visualize viable neurons under the light microscope. Images were taken using the software Optika Vision Lite 2.1 at 4× and 40× magnification. Neuronal density was quantified in the molecular, extra granular and pyramidal, inner granular, ganglionic, and multiform layers of the motor and somatosensory cortex by averaging count from four fields of view per section (7500 µm^3^ area) and the Cornu Ammonis (CA1, CA2, and CA3) and dentate gyrus (DG) in the hippocampus by averaging counts from three fields of view per section (2450 µm^3^ area).

### 2.9. GC/MS Analysis for Metabolomic Profiling of Brain Tissues

#### 2.9.1. Sample Preparation

GC/MS analysis was performed using brain samples of 2 animals from each group. A 10mg representative aliquot, uniformly obtained from the harvested whole-brain tissue of each animal, was transferred into a 1.5 mL Eppendorf tube and homogenized for 2–3 min in 1000 μL of solvent mixture (MeOH:H_2_O:CHCl_3_ = 5:2:2). Then 100 μL of Ribitol solution in water (0.4 mg/mL) as internal standard was added into each sample followed by the addition of 800 μL of methanol. Samples were vortexed for one minute each and then shaken for 30 min at 700 rpm at 15 °C. Samples were later centrifuged for 10 min at 10,000 rpm followed by Speed Vac under vacuum for 3 or 4 h till they were completely dry. A total of 40 μL of methoxyamine hydrochloric acid (HCL) was added to each vial and incubated at room temperature overnight (16 h). A total of 50 μL MSTFA derivatizing agent was added to each vial and incubated at 60 °C for 2 h. At the end, GC/MS was performed on a 7890A gas chromatography (Agilent technologies, Santa Clara, CA, USA) equipped with an Agilent Technology GC autosampler 120 (PAL LHX-AG12) and coupled to an Agilent 7000 Triple Quad system (Agilent technologies, Santa Clara, CA, USA). An HP-5MS 30 m-250 mm (i.d.) fused-silica capillary column (Agilent J&W Scientific, Folsom, CA, USA), chemically bonded with a 5% diphenyl 95% dimethylpolysiloxane cross-linked stationary phase (0.25 mm film thickness), was used. Helium was used as the carrier gas at 1.0 mL min and the sample was injected in splitless mode. The injector and source temperatures were 250 °C. The oven temperature was initially maintained at 40 °C and was then increased at 10 °C min to 300 °C and retained at 300 °C for 9 min. In post run, temperature was further increased to 305 °C for the remaining 1 min. Electron ionization (EI) is used as an ionization source for the GC/MS analysis at 70 eV. Data is acquired in the full scan mode from *m*/*z* 50 to 700 with a scan time of 0.5 s. Three replicates of each sample were performed then blank (hexane) was run in between each sample to remove any contamination. Perfluorotributylamine (PFTBA) was used for mass calibration. Data processing was performed by using the Agilent Mass Hunter Qualitative Analysis (version B.04.00).

The chromatographic deconvolution parameters on Mass Hunter are set as follows: SNR threshold 10.0, RT window size factor: 100.0, left *m*/*z* delta: 0.3 (amu), right *m*/*z* delta: 0.7 (amu), excluded *m*/*z* 28 (amu), sharpness threshold: 25.0% and chromatogram deconvolution compound filter parameter as relative area 1.0% of the largest peak, and absolute area 5000 counts. Putatively the identity of GC/MS-detected peaks was established by comparing the mass spectra of the peaks with those available in the NIST mass spectral (Wiley registered library 11).

#### 2.9.2. Significance and Fold Change Analysis

Significance and fold change analysis was performed after comparing the mass spectra of the obtained peaks with the data already available in NIST mass spectral library. This is level 2 of the Metabolomics Standard Initiative for Identification.

#### 2.9.3. Principal Component Analysis

The dimensionality of the dataset was reduced via principal component analysis (PCA), which also highlighted the variance between all experimental groups. After this, discrimination analysis was performed to get a clearer understanding of the most significantly variated metabolites.

#### 2.9.4. Discrimination Analysis

Multivariate data analysis was performed to authenticate the GC/MS analysis differentiating various groups. One of the supervised multivariate data analyses is PLS-DA (Partial Least Squares-Discriminant Analysis) that helps in establishing better and precise models for discrimination between different groups. The variable importance in projection (VIP) scores were calculated to identify key metabolites contributing to group discrimination. Only metabolites with VIP scores > 1 were considered significant for group separation.

#### 2.9.5. Pathway Analysis and Heatmap

Pathway analysis was done via Kyoto Encyclopedia of Genes and Genomes (KEGG) pathway database URL: https://www.genome.jp/kegg/pathway.html (assessed on 28 November 2024) to assess the roles of various metabolites identified in GC/MS analysis.

### 2.10. Statistical Analyses

The data were analyzed, and graphs were plotted using GraphPad Prism (version 5.01). All statistical analyses were done by one-way analysis of variance (ANOVA) followed by the Bonferroni post hoc test. The magnitude of differences (variance ratio) is reported via F-statistics (F_(dfn,dfd)_) and precise *p* values to illustrate the effect size of SEGE intervention compared to the Met + HFD group. Data with *p* value < 0.05 were considered significant and presented as mean ± SEM. For the sake of simplicity, only the significant difference of Met + HFD group is shown with the rest of the groups.

## 3. Results

### 3.1. SEGE Restores Expression of Synaptic Plasticity Markers

**Figure 4:**
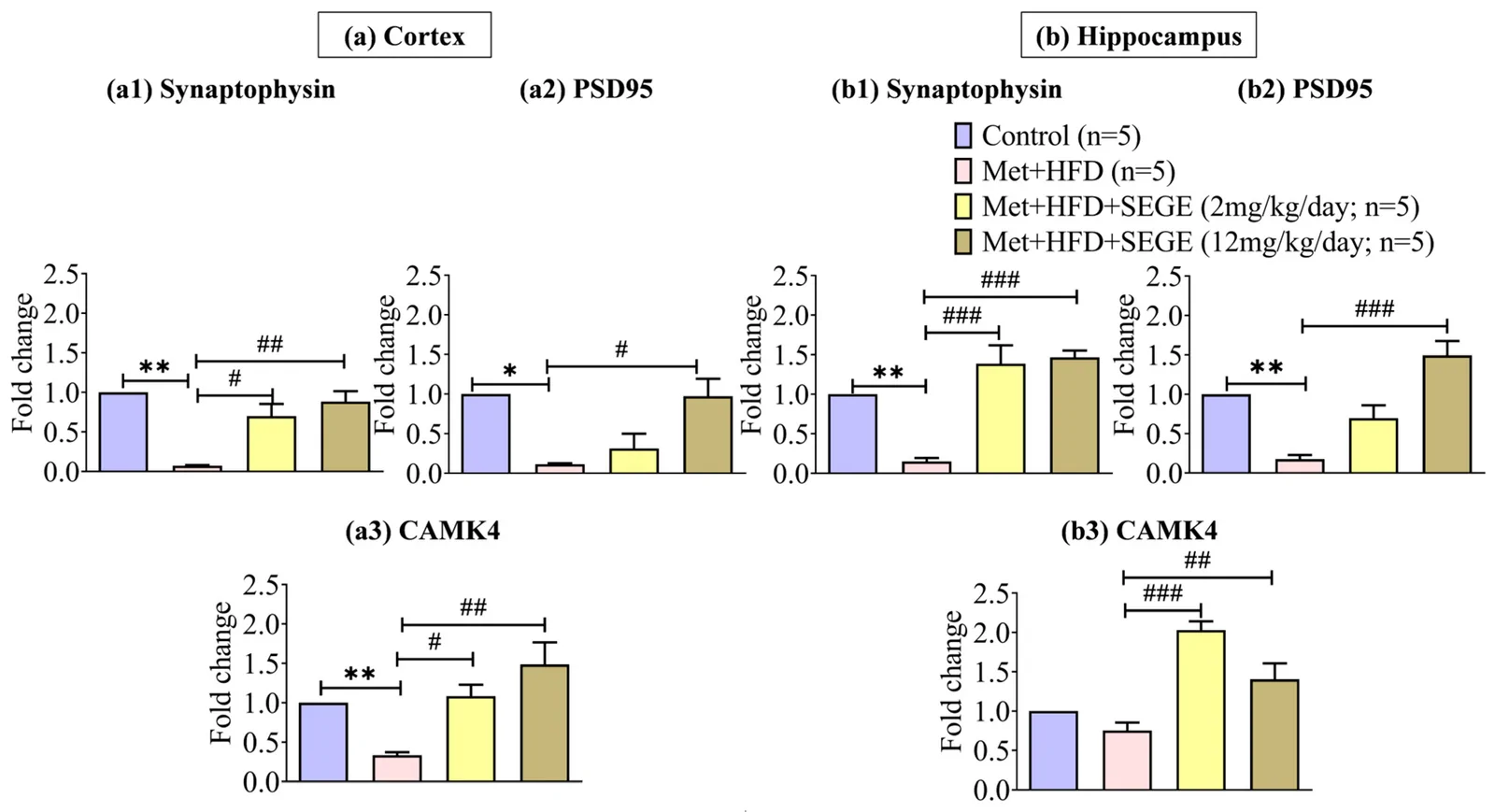
Relative expression of synaptic plasticity genes in (**a**) cortex and (**b**) hippocampus. Graphs showing (**a1**) levels of synaptophysin, (**a2**) levels of *PSD95*, and (**a3**) levels of *CAMK-4* in the cortex and (**b1**) levels of synaptophysin, (**b2**) levels of *PSD95*, and (**b3**) levels of *CAMK-4* in the hippocampus in control, Met + HFD, Met + HFD + SEGE (2 mg/kg/day), and Met + HFD + SEGE (12 mg/kg/day) groups. Statistical significance was assessed via one-way ANOVA followed by Bonferroni’s post hoc test. Significant values are represented as * = *p* < 0.05, ** = *p* < 0.01, for difference between Met + HFD and Control, and as # = *p* < 0.05, ## = *p* < 0.01, ### = *p* < 0.001 for difference between Met + HFD and SEGE treated groups. Error bars are represented as mean ± SEM.

To evaluate the combined metals and HFD toxicity, mRNA expression levels of Synaptophysin, *PSD95*, and *CAMK-4* were assessed in the cortex and hippocampus. In the cortex, Met + HFD group showed a significant downregulation of Synaptophysin (one-way ANOVA; F (3, 8) = 16.69; 0.07 ± 0.01; *p* < 0.01), *PSD95* (F (3, 8) = 9.894; 0.11 ± 0.02; *p* < 0.05), and *CAMK-4* (F (3,8) = 9.15; 0.33 ± 0.06; *p* < 0.01) compared to control (1.0 ± 0.0). Treatment with SEGE at high dose (12 mg/kg/day) significantly restored the expression of all three markers (Synaptophysin: 0.88 ± 0.22; *p* < 0.01, *PSD95*: 0.97 ± 0.38; *p* < 0.05, and *CAMK-4*: 1.48 ± 0.48; *p* < 0.01; Figure 4(a1–a3)).

In the hippocampus, a similar pattern was observed. Met + HFD group showed severely downregulated Synaptophysin (one-way ANOVA; F (3, 8) = 22.92; 0.15 ± 0.07; *p* < 0.01) and *PSD95* (F (3, 8) = 19.58; 0.17 ± 0.08; *p* < 0.01). Interestingly, the levels of *CAMK-4* remained unaltered in the Met + HFD group compared to the control group. SEGE treatment at both the doses (2 mg/kg/day and 12 mg/kg/day) upregulated Synaptophysin (1.38 ± 0.40 and 1.46 ± 0.14, respectively; *p* < 0.001) and *CAMK-4* (2.03 ± 0.19; *p* < 0.001; 1.4 ± 0.35; *p* < 0.01, respectively) beyond control levels (1.0 ± 0.0). PSD95 expression was rescued following treatment with the high dose (12 mg/kg/day) SEGE (1.4 ± 0.31; *p* < 0.001; Figure 4(b1–b3)).

### 3.2. SEGE Modulates Cholinergic Receptor Subunits and ChAT Expression

**Figure 5:**
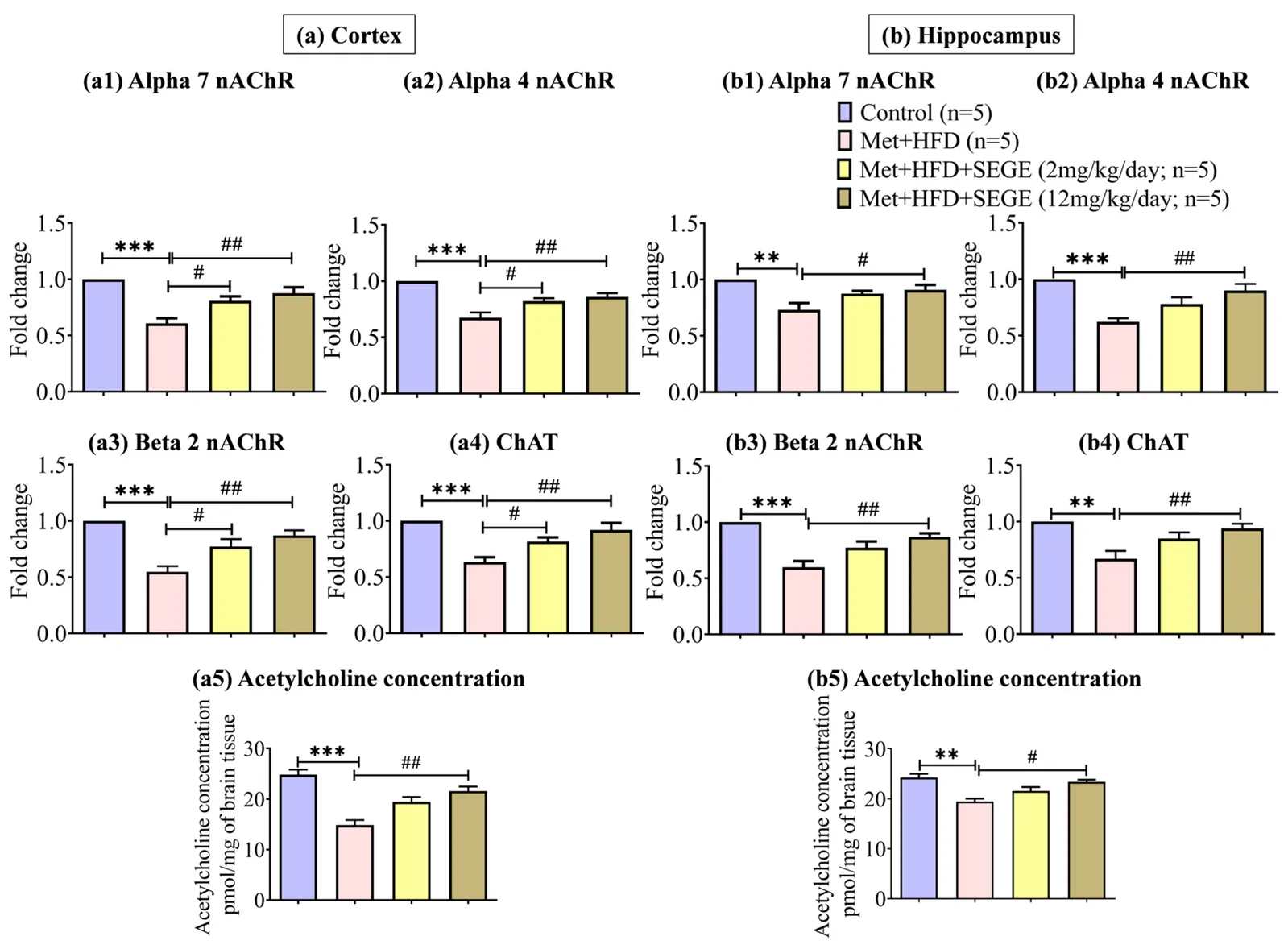
Relative expression of cholinergic receptors’ genes, *ChAT* gene and acetylcholine concentration in (**a**) cortex and (**b**) hippocampus. Graphs showing (**a1**) levels of *α*7*nAChR* in cortex, (**a2**) levels of *α4nAChR*, (**a3**) levels of *β2nAChR*, (**a4**) levels of *ChAT*, and (**a5**) ACh concentration in cortex, and (**b1**) levels of *α7nAChR*, (**b2**) levels of *α4nAChR*, (**b3**) levels of *β2nAChR*, (**b4**) levels of *ChAT*, and (**b5**) ACh concentration in hippocampus in control, Met + HFD, Met + HFD + SEGE (2 mg/kg/day), and Met + HFD + SEGE (12 mg/kg/day) groups. Statistical significance was assessed via one-way ANOVA followed by Bonferroni’s post hoc test. Significant values are represented as ** = *p* < 0.01, *** = *p* < 0.001 for difference between Met + HFD and Control, and as # = *p* < 0.05, ## = *p* < 0.01 for difference between Met + HFD and SEGE treated groups. Error bars are represented as mean ± SEM.

The expression of nicotinic acetylcholine receptor (*nAChR*) subunits (α7, α4, and β2) and the ACh synthesis enzyme Choline Acetyltransferase (*ChAT*) was evaluated to assess cholinergic functions. In the cortex, the Met + HFD group showed widespread suppression of cholinergic genes, with significant decreases in *α7nAChR* (one-way ANOVA; F (3, 16) = 16.65; 0.61 ± 0.05; *p* < 0.001), α1nAChR (F (3, 16) = 18.47; 0.67 ± 0.05; *p* < 0.001), *β2nAChR* (F (3, 16) = 16.49; 0.59 ± 0.05; *p* < 0.001), and ChAT (F (3, 16) = 14.26; 0.63 ± 0.04; *p* < 0.001) compared to control (1.0 ± 0.0). SEGE treatment at high dose (12 mg/kg/day) significantly reversed these deficits for all measured genes (*p* < 0.01). The low dose (2 mg/kg/kg) also provided significant protection (*p* < 0.05), upregulating all four markers compared to Met + HFD group (Figure 5(a1–a4)).

Similarly, in the hippocampus, Met + HFD downregulated all receptor subunits (*α7nAChR*: one-way ANOVA; F (3, 16) = 8.08; 0.73 ± 0.06; *p* < 0.01; *α4nAChR*: F (3, 16) = 13.36; 0.62 ± 0.03; *p* < 0.001; *β2nAChR*: F (3, 16) = 16.29; 0.59 ± 0.05; *p* < 0.001; *ChAT*: F (3, 16) = 8.70; 0.67 ± 0.07; *p* < 0.01) compared to the control (1.0 ± 0.0). In this region, the protective effect of SEGE was dose-dependent; only the high dose (12 mg/kg/day) significantly restored the expression levels of *α7nAChR* (0.91 ± 0.05; *p* < 0.05), *α4nAChR* (0.9 ± 0.13; *p* < 0.01), *β2nAChR* (0.87 ± 0.03; *p* < 0.01), and *ChAT* (0.94 ± 0.04; *p* < 0.01). The low dose (2 mg/kg/day) did not yield a statistically significant improvement in hippocampal cholinergic gene expression (Figure 5(b1–b4)).

### 3.3. Recovery of ACh Levels

Consistent with the gene expression data, the biochemical analysis revealed a sharp decline in the ACh levels in the cortex of Met + HFD group (one-way ANOVA; F (3, 8) = 19.64; 14.86 ± 1.0; *p* < 0.001) compared to the control group (24.88 ± 0.96). Treatment with SEGE at high dose (12 mg/kg/day) significantly restored the ACh levels (21.62 ± 0.87; *p* < 0.01), while low dose (2 mg/kg/day) did not show effect (Figure 5(a5)).

In the hippocampus, ACh level was also reduced significantly in Met + HFD (one-way ANOVA; F (3, 8) = 10.82; 19.47 ± 0.58; *p* < 0.01) compared to the control (24.29 ± 0.75), which was again significantly restored by SEGE treatment at high dose only (12 mg/kg/day; 23.42 ± 0.43; *p* < 0.05; Figure 5(b5)).

### 3.4. SEGE Treatment Attenuates Neurodegeneration in the Hippocampus

**Figure 6:**
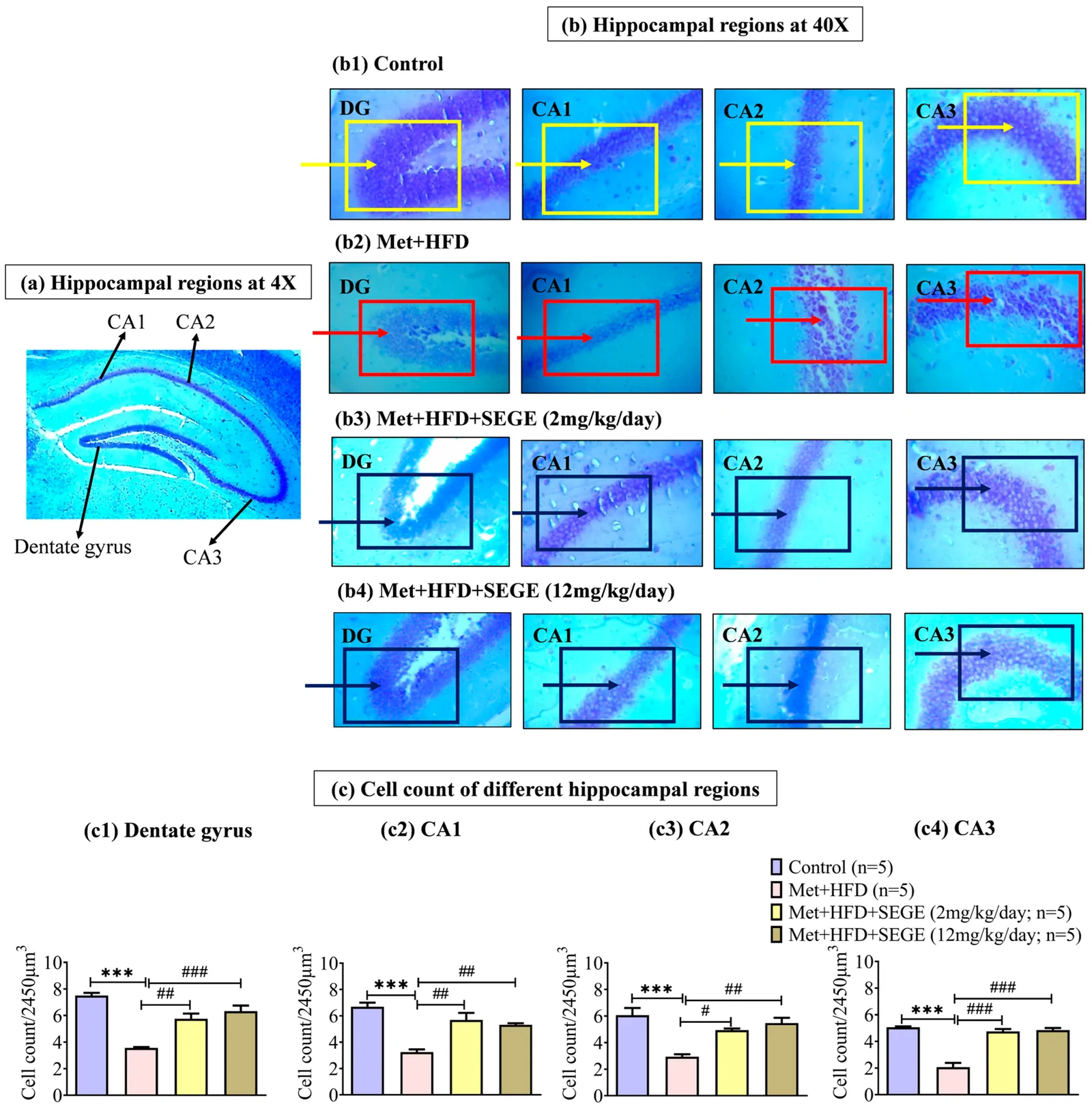
Histology and cell count of different hippocampal regions. Histology of different hippocampal regions at (**a**) 4× and histology at 40× in (**b1**) Control, (**b2**) Met + HFD, (**b3**) Met + HFD + SEGE (2 mg/kg/day), and (**b4**) Met + HFD + SEGE (12 mg/kg/day). Graphs showing cell count of (**c1**) Dentate gyrus (DG), (**c2**) CA1, (**c3**) CA2, and (**c4**) CA3 in hippocampus in control, Met + HFD, Met + HFD + SEGE (2 mg/kg/day), and Met + HFD + SEGE (12 mg/kg/day) groups. The dense neuronal areas in hippocampal regions of Control are indicated by yellow arrows in (**b1**) while the neuronal loss in Met + HFD is indicated by red arrows in (**b2**). The dark blue arrows in (**b3**,**b4**) indicate the dense neuronal areas in Met + HFD + SEGE (2 mg/kg/day) and Met + HFD + SEGE (12 mg/kg/day), respectively. Statistical significance was assessed via one-way ANOVA followed by Bonferroni’s post hoc test. Significant values are represented as *** = *p* < 0.001 for difference between Met + HFD and Control, and as # = *p* < 0.05, ## = *p* < 0.01, ### = *p* < 0.001 for difference between Met + HFD and SEGE treated groups. Error bars are represented as mean ± SEM.

In histological assessment, Met + HFD group showed marked neurodegeneration (Figure 6(a,b1–b4)) and significantly reduced cell counts across all sub-regions: DG (one-way ANOVA; F (3, 12) = 28.37; 3.56 ± 0.06; *p* < 0.001), CA1 (F (3, 12) = 19.02; 3.25 ± 0.20; *p* < 0.001), CA2 (F (3, 12) = 14.81; 2.94 ± 1.87: *p* < 0.001), and CA3 (F (3, 12) = 48.08; 2.06 ± 0.32; *p* < 0.001).

SEGE treatment provided robust neuroprotection. Both the doses showed preserved neuronal density and rescued the neuronal count in the DG (5.75 ± 0.40; *p* < 0.01; 6.33 ± 0.43; *p* < 0.001, respectively), CA1 (5.69 ± 0.53; 5.31 ± 0.12, respectively; *p* < 0.01), CA2 (4.94 ± 0.12; *p* < 0.05; 5.48 ± 0.39; *p* < 0.01, respectively) and CA3 (4.75 ± 0.18; 4.75 ± 0.18, respectively; *p* < 0.001) sub-regions compared to Met + HFD group (Figure 6(c1–c4)).

### 3.5. SEGE Treatment Attenuates Neurodegeneration in the Cortex

**Figure 7:**
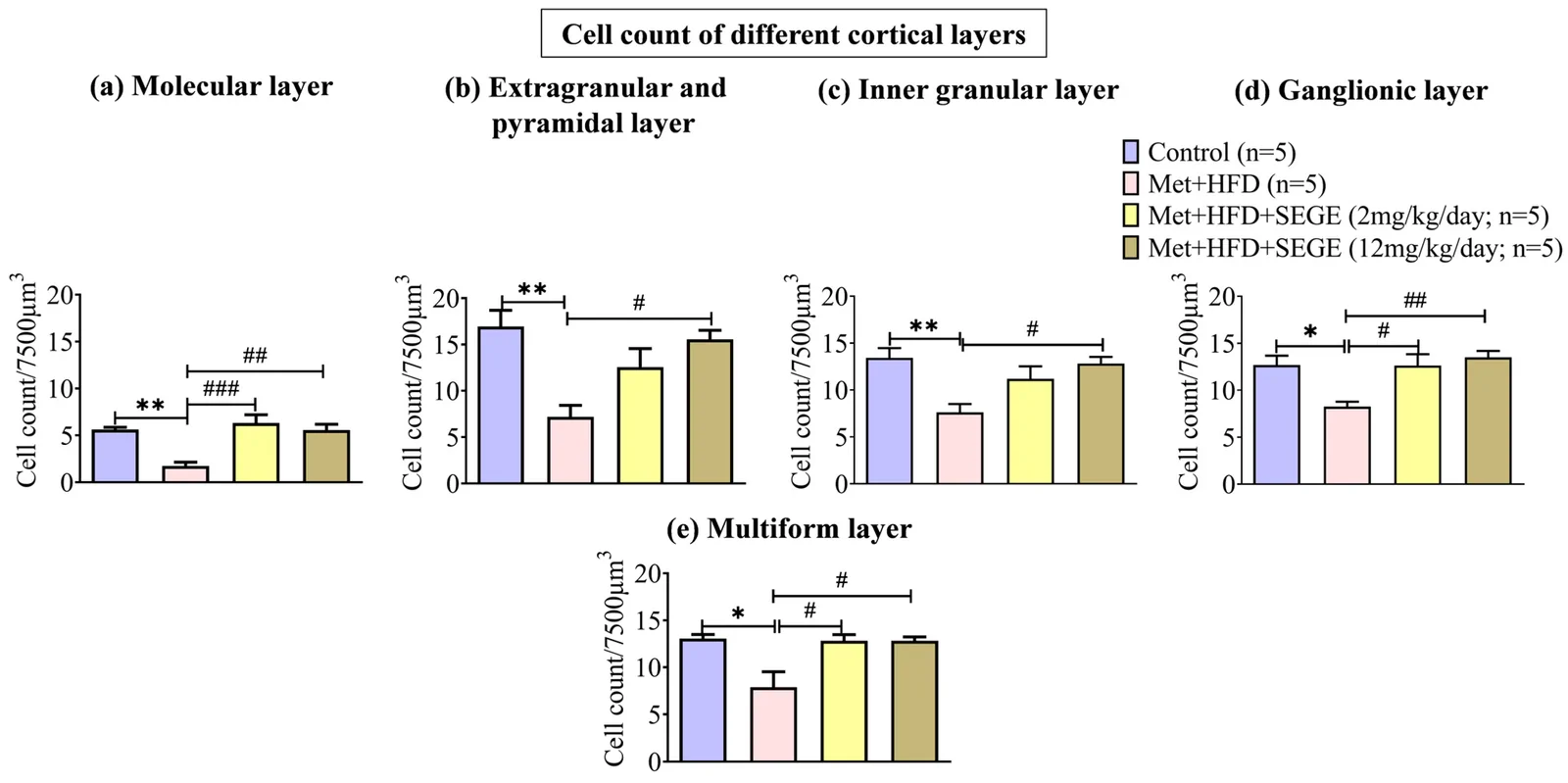
Cell count of different cortical layers. Graphs showing cell count of (**a**) molecular layer, (**b**) extra-granular and pyramidal layer, (**c**) inner granular layer, (**d**) ganglionic layer, and (**e**) multiform layer in cortex of control, Met + HFD, Met + HFD + SEGE (2 mg/kg/day), and Met + HFD + SEGE (12 mg/kg/day) groups. Statistical significance was assessed via one-way ANOVA followed by Bonferroni’s post hoc test. Significant values are represented as * = *p* < 0.05, ** = *p* < 0.01 for difference between Met + HFD and Control, and as # = *p* < 0.05, ## = *p* < 0.01, ### = *p* < 0.001 for difference between Met + HFD and SEGE treated groups. Error bars are represented as mean ± SEM.

Neuronal counting was also done in cortical brain tissues of mice and there was a significant decline in the cell count of molecular layer in Met + HFD (one-way ANOVA; F (3, 12) = 12.44; 1.75 ± 0.39) compared to control (5.62 ± 0.24; *p* < 0.01), Met + HFD + SEGE (2 mg/kg/day; 6.31 ± 0.88; *p* < 0.001), and Met + HFD + SEGE (12 mg/kg/day; 5.56 ± 0.62; *p* < 0.01; Figure 7a). Extra-granular and pyramidal layer also showed a significantly reduced cell count in Met + HFD (one-way ANOVA; F (3, 12) = 7.84; 7.18 ± 1.24) compared to the control (16.94 ± 1.75; *p* < 0.01) and Met + HFD + SEGE (12 mg/kg/day; 15.56 ± 0.96; *p* < 0.05; Figure 7b). A similar trend was observed in the inner granular layer showing a decrease in Met + HFD (one-way ANOVA; F (3, 12) = 6.55; 7.61 ± 0.89) compared to the control (13.44 ± 1.03; *p* < 0.01) and Met + HFD + SEGE (12 mg/kg/day; 12.81 ± 0.71; *p* < 0.05; Figure 7c). The downward trend remained the same in the cell count of Met + HFD in ganglionic and multiform layers. In ganglionic layer, the cell count was reduced significantly in Met + HFD (one-way ANOVA; F (3, 12) = 7.20; 8.25 ± 0.53) compared to control (12.69 ± 0.98; *p* < 0.05), Met + HFD + SEGE (2 mg/kg/day; 12.63 ± 1.21; *p* < 0.05) and Met + HFD + SEGE (12 mg/kg/day; 13.50 ± 0.68; *p* < 0.01; Figure 7d). Finally, in the multiform layer, the cell count was significantly reduced in Met + HFD (one-way ANOVA; F (3, 12) = 7.18; 7.86 ± 1.64) compared to the control (13.64 ± 0.43; *p* < 0.05), Met + HFD + SEGE (2 mg/kg/day; 12.81 ± 0.66; *p* < 0.05) and Met + HFD + SEGE (12 mg/kg/day; 12.81 ± 0.43; *p* < 0.05; Figure 7e).

### 3.6. Effects of Metals, HFD, and SEGE on Brain’s Metabolic Profile

#### 3.6.1. Significance and Fold Change Analysis

In total, 466 metabolites were identified, out of which 72 metabolites were found significantly differentiated. These 72 entities were putatively identified with a 70% similarity index (no other entity was identified at this similarity index).

#### 3.6.2. Principal Component Analysis

**Figure 8:**
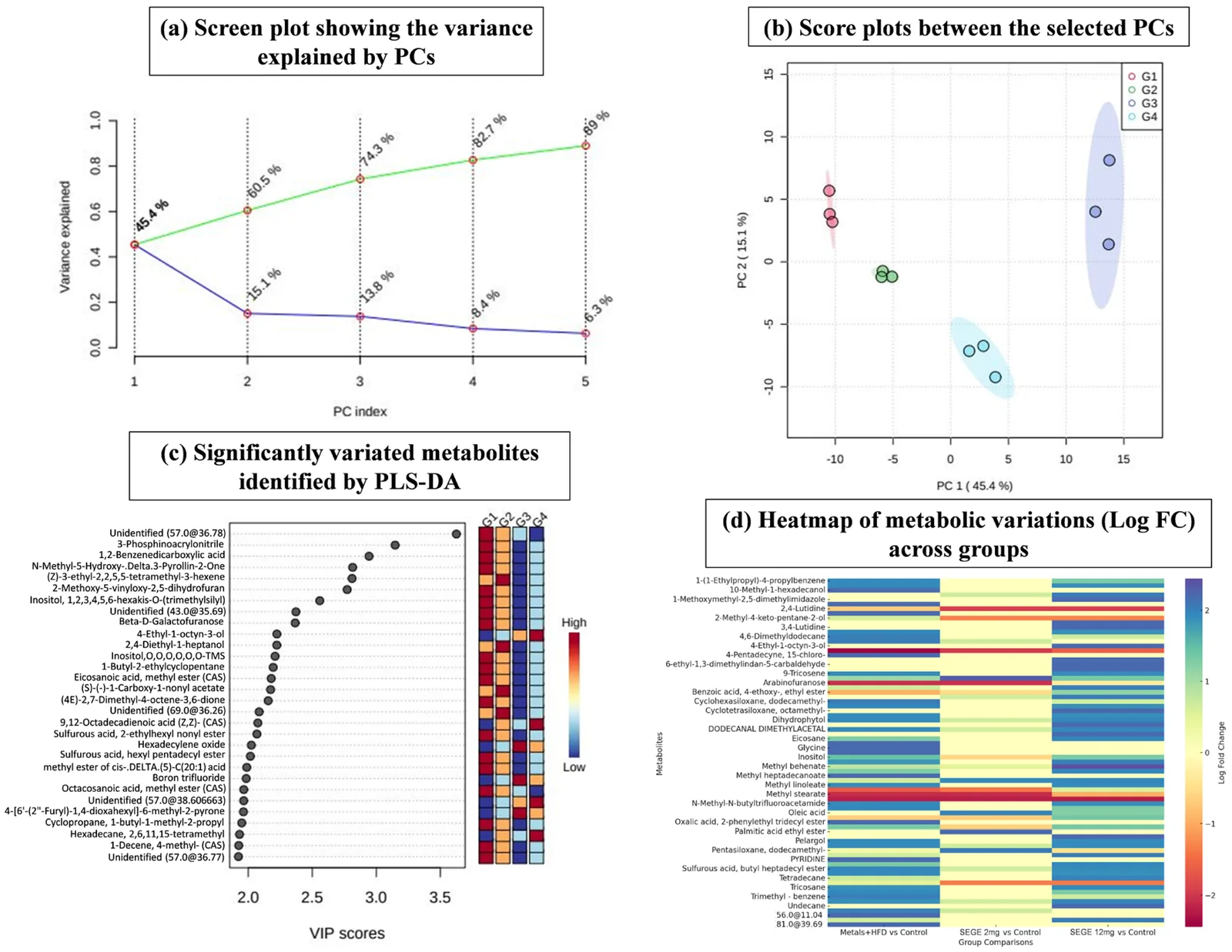
GC/MS analysis of the metabolites present in the brain. (**a**) Screen plot shows the variance explained by principal components (PCs). The green line on top shows the accumulated variance explained; the blue line underneath shows the variance explained by individual PC. (**b**) Scores plot between the selected PCs. The explained variances are shown in brackets. (**c**) Significantly variated metabolites identified by PLS-DA (VIP Scores). The colored boxes on the right indicate the relative concentrations of the corresponding metabolite in each group under study and (**d**) heatmap of metabolic variations (Log FC) across groups. The colored scale indicating (from 2 to −2) from highest variation to highest non-variation, respectively. Groups are control as G1, Met + HFD as G2, Met + HFD + SEGE (2 mg/kg/day) as G3, and Met + HFD + SEGE (12 mg/kg/day) as G4, respectively. The metabolites along with their associated pathway are also given in Table S1.

The first two PCs, i.e., PC1 (45.4%) and PC2 (15.1%), accounted for a total variance of 60.5%, indicating that the majority of the variation in the dataset can be described by these two components. This also indicates that the same two components can be used to visualize group differences. Both the screen plot and scores plot in Figure 8a,b showed a clear group separation with Met + HFD group showing a cluster far away from the control group, while the SEGE (12 mg/kg/day) treated group showed a cluster near to the control group. The group treated with SEGE (2 mg/kg/day), however, showed a cluster in between control group and Met + HFD group.

#### 3.6.3. Discrimination Analysis

In PLS-DA analysis, 30 key metabolites were identified to be significantly differentiated between control, Met + HFD, Met + HFD + SEGE (2 mg/kg/day), and Met + HFD + SEGE (12 mg/kg/day; Figure 8c).

#### 3.6.4. Pathway Analysis and Heatmap

Most of the metabolites identified were found to be involved in amino acids metabolism pathways, lipids metabolism pathways, neuronal signaling, neurotransmitters synthesis, cell membrane structures, and inflammatory pathways. The pathway and function of each of the 72 significantly differentiated metabolites is mentioned in Table S1 and the heatmap of the significantly variated and non-variated metabolites is given in Figure 8d.

Specifically, the data revealed that the Met + HFD group exhibited a marked upregulation of metabolites linked to fatty acid and lipid metabolism (e.g., 10-Methyl-1-hexadecanol, 2,3,6-Trimethyloctance, 2-Methyl-4-keto-pentane-2-ol, Methyl heptadecanoate, and Cholestene) and of the inhibitory neurotransmitter (Glycine), alongside a significant downregulation of compounds involved in stimulatory neurotransmitter pathways (e.g., 1-Methoxy-9-octadecene, 2,4-Lutidine, 3,4-Lutidine, and 1-Methoxymethyl-2,5-dimethylimidazole). Treatment with SEGE, particularly at the higher dose of 12 mg/kg/day, successfully reversed these trends. The 12 mg/kg/day SEGE intervention effectively normalized the expression of these key metabolites, balancing energy metabolism and neurotransmission profiles closer to the baseline levels observed in the control group.

## 4. Discussion

The convergence of industrial pollution and the global shift toward the western, high-fat diets represents a complex toxicological challenge [54,55,56,57]. While heavy metals are well-reported neurotoxicants that induce oxidative stress and mitochondrial dysfunction [58,59,60] and HFDs reportedly impair BBB integrity and induce systemic inflammation [61], their combined effects remain largely under-characterized. This study validated a double-hit model in which chronic co-exposure to a metal mixture (As, Pb, and Al) and HFD governed severe cholinergic and synaptic dysfunctions. Importantly, the study also demonstrated that a defined SEGE significantly ameliorates these neuropathological deficits, offering a translatable dietary intervention.

Metals and HFD are reported to cause disrupted synaptic plasticity by increasing ROS, obesity, and insulin resistance [62,63,64]. We have also previously reported high levels of heavy metals in cognitively impaired patients in Pakistan [7]. In the present study, the Met + HFD group showed significant downregulation of synaptophysin and *PSD95* genes in the hippocampus and cortex. This aligns with previous findings that metals like As and Pb disrupt synaptic proteins via ROS-mediated damage, while HFD impaired hippocampal plasticity through insulin-resistance pathways [43,44,45]. Treatment with SEGE restored these markers, likely due to the antioxidant potential of 6-SG, which neutralizes the ROS responsible for synaptic degradation [46]. Interestingly, while *CAMK-4* expression was suppressed in the cortex, it remained unaltered in the hippocampus of the Met + HFD exposed group. However, SEGE significantly upregulated *CAMK-4* in the hippocampus beyond control levels. This suggests that SEGE may not only protect against toxicity but also potentiate baseline synaptic signaling pathways, a property valuable for neuroprotection [46]. This overall upregulation of synaptic plasticity genes in SEGE-treated groups is like previously reported effects of SG on neurotrophic genes like *BDNF* and Cyclic Adenosine Monophosphate Response Element Binding Protein (*CREB*) via enhanced phosphorylation [65], validating SEGE’s therapeutic potential in neuropathology. Additionally, metals and HFD reduced the expression of cholinergic receptors [66,67], including *nAChRs* subunits (α7, α4, and β2) and *ChAT*, in both cortex and hippocampus. SEGE restored the expression of these genes, with better results at the higher dose (12 mg/kg/day). This suggests dose-dependent effects on the cholinergic system, which is consistent with the findings in a Parkinson’s disease model [32]. Although the correlation between gene expression and corresponding protein is a debatable topic but some previous studies have shown a strong positive correlation between mRNA and protein [68], further validating this data.

Cholinergic dysfunction is a hallmark of neurodegenerative diseases like Alzheimer’s disease [47] but very few studies have shown the effects of metals and HFD specifically on hippocampus- and cortex-associated cognition. In the current study, neurochemical analysis revealed reduced *nAChR* subunits (α7, α4, and β2) and ACh level in these regions in Met + HFD group, likely due to decreased ChAT level, an enzyme critical for ACh synthesis. Increased HFD intake reduces *ChAT*, increases AChE activity, and elevates ROS levels, leading to cholinergic dysfunction [56,69], dementia, and cognitive decline [70]. In contrast, treatment with both doses of SEGE showed increased ACh levels, indicating that SEGE may help restore normal ACh levels and may rescue cholinergic hypofunction [71], particularly at the higher dose (12 mg/kg/day). The restoration of *α7nAChR* is particularly significant, as this receptor modulates neuroinflammation [32]. 6-SG has been previously reported to activate anti-inflammatory pathways [63]; therefore, the present study suggests that this mechanism extends to the protection of vulnerable cholinergic neurons in a complex toxicity model.

Metals and HFD have also been linked to neuroinflammation and neurodegeneration [70,72,73], characterized by the neuronal death [74]. In this study, histological analysis confirmed that the biochemical deficits translated into structural damages. Met + HFD group displayed a reduced neuronal count in cortical layers and a shrunken hippocampus. Both SEGE-treated groups showed reduced neurodegeneration, with the higher dose showing better results. Further high-resolution studies are required to examine anatomical detail. Reduced neuronal count in Met + HFD group, consistent with a previous study [21], may be due to inflammatory pathways and oxidative stress due to metals and HFD [75,76]. These results showed that SEGE treatment caused beneficial effects through multiple pathways [77]. Overall, the histological analysis strengthened the data of cholinergic and synaptic assessments.

The metabolic pathways in the body are made up by the functional association of groups of proteins, interacting genes, causing production of various metabolites during the processes of cellular function. Any dysregulation in these pathways/networks can lead to severe metabolic alterations causing various diseases [78,79]. All four groups were assessed for metabolites by GC/MS followed by extensive chemometric analysis. The metabolic analysis and PLS-DA revealed significant changes in lipid metabolism, oxidative stress markers, and neurotransmitter-related metabolites across experimental groups, consistent with the results from other experiments in the study. The Met + HFD group showed an upregulation of metabolites linked to fatty acid metabolism (e.g., 10-Methyl-1-hexadecanol, 2,3,6-Trimethyloctance, 2-Methyl-4-keto-pentane-2-ol, Methyl heptadecanoate, and Cholestene), upregulation of inhibitory neurotransmitter (Glycine), and a downregulation of compounds involved in stimulatory neurotransmitter pathways (e.g., 1-Methoxy-9-octadecene, 2,4-Lutidine, 3,4-Lutidine, and 1-Methoxymethyl-2,5-dimethylimidazole), suggesting increased lipid dysregulation and neurotransmitter imbalance, both of which are known contributors in various neurological disorders [80,81]. The biological significance of these specific alterations is directly linked to the dual-hit model; the profound lipid dysregulation primarily reflects the systemic lipotoxicity of the HFD, while the suppressed neurotransmitter precursors mirror the energetic and oxidative collapse induced by heavy metal bioaccumulation [70,72,73]. However, treatment with SEGE, particularly at the high dose, indicated a mitigation of these effects by acting as a robust metabolic buffer; its active compounds neutralize metal-induced oxidative stress and restore lipid homeostasis, thereby halting the downstream cascade that leads to cholinergic and synaptic failure [46,63].

Taking together these associative findings, we propose a cohesive mechanistic cascade underlying the observed neurotoxicity. The initial systemic metabolic disruptions and cellular energy deficits, as revealed by the GC/MS metabolic profiling, appear to serve as primary upstream catalysts. This metabolic failure may drive severe oxidative stress, which subsequently compromises cholinergic integrity. The resulting cholinergic depletion and sustained metabolic stress directly precipitate structural breakdown of synaptic plasticity markers (Synaptophysin, *PSD95*, and *CAMK-4*) and the widespread neuronal loss observed in the cortical and hippocampal layers. SEGE’s efficacy may therefore lie in its multi-targeted ability to intercept this cascade at the systemic metabolic level, thereby preserving cholinergic function and rescuing downstream synaptic architecture.

Various health issues, especially neurological issues are dramatically increasing in human beings due to heavy metals [7,82] in combination with HFD [56]. The current study has uniquely assessed the effects of heavy metals exposure in combination with HFD on synaptic plasticity and cholinergic systems leading to neuropathology, which may be the underlying cause of cognitive impairment. It is important to note the deliberate design parameters of the current study, which are, on the one hand, a limitation of the study, while, on the other, a strength of it. The study specifically utilized a “double-hit” model to mimic complex, real-world environmental and dietary co-exposures rather than isolating individual variables. The absence of single-exposure control groups (heavy metals alone and HFD alone) precludes the statistical isolation of each factor’s individual neurotoxic contribution compared to the combined Met + HFD model. However, given the well-documented individual neuropathological effects of both heavy metals’ toxicity and HFD, prioritizing the combined group allowed us to strictly focus on the synergistic pathology of the modern exposome and evaluate the robust efficacy of SEGE against compound biological stress, while strictly adhering to the ethical principles of reducing animal use in experimental research. Moreover, the study utilized a whole extract rather than a pure isolate, which, on the one hand, is a limitation but, on the other hand, is a strength. As the HPLC characterization confirmed the presence of both 6-SG (major component) and 6-GN (minor component), it can be concluded that whole-extract SEGE appears to provide broader protection than isolated compounds; however, the synergy between these constituents is a hypothesis for future investigation rather than an experimental conclusion. This whole-food approach closely mimics the dietary patterns, providing proof of concept for translational relevance of the findings. But the study has some limitations; although many of the results showed that SEGE at a high dose is more effective in controlling Met + HFD induced neuropathology, many other results have shown a mixed trend for both SEGE doses (2 mg/kg/day and 12 mg/kg/day), suggesting further evaluation. Moreover, while the current study relies on mRNA quantification, and future studies incorporating protein-level validation and direct pharmacokinetic assessment are warranted, the observed downregulation of these synaptic markers strongly correlates with functional cognitive impairments typically seen in double-hit toxicity models [22,83]. Additionally, the GC/MS metabolic profiling was conducted on a relatively small sample size (*n* = 2 per group); therefore, these specific metabolic shifts should be interpreted cautiously, warranting validation in larger future cohorts, along with independent functional assays to establish direct causality.

## 5. Conclusions

This study has well-established multiple underlying mechanisms of neuropathology in the combination of heavy metals with HFD exposure to animals. Meanwhile, the neuroprotective potential of SEGE is elaborated in Met + HFD induced neuropathology along with brain’s metabolic profile in a mouse model. The results have shown that Met + HFD co-administration resulted in reduced neuronal number, decreased level of acetylcholine and disruption in synaptic plasticity and major metabolic pathways. Understanding the role of metals in combination with HFD in inducing various neuropathies and cognitive dysfunctions may be helpful in developing better preventive tools for neurological disorders. Similarly, understanding the role of SEGE in prevention of cognitive decline may also be helpful in utilizing it (or similar natural plant extracts) as an effective dietary intervention either for the control or for the treatment of many neurological diseases in the future.

## Figures and Tables

**Table 1:** Animal model development.

No.	Group Name	Animal Model Development	Total Mice in Each Group
1	Control group	Distilled drinking water and normal feed	10
2	Met + HFD group	75 mg/kg metals (As, Pb, and Al; 25 mg/kg each) in drinking water + 40% HFD (No SEGE treatment)	10
3	Met + HFD + SEGE (2 mg/kg/day) group	75 mg/kg metals (As, Pb, and Al; 25 mg/kg each) in drinking water + 40% HFD + 2 mg/kg SEGE mixed in HFD	10
4	Met + HFD + SEGE (12 mg/kg/day) group	75 mg/kg metals (As, Pb, and Al; 25 mg/kg each) in drinking water + 40% HFD + 12 mg/kg SEGE mixed in HFD	10
Total no. of mice			40

**Table 2:** Real-time PCR primers and their annealing temperatures.

No.	Gene	Primer Sequence 5’ to 3’	Annealing Temp.
1	Glyceraldehyde-3-Phosphate (*GAPDH*) F primer *GAPDH* R primer	CCTGGCCAAGGTCATCCAT GTCATGAGCCCTTCCACGAT	60 °C
2	Calcium/Calmodulin Dependent Protein Kinase 4 (*CAMK4*) F primer *CAMK4* R primer	CGAAGATGCTCAAAGTCACGG ACTCCACCTCGAAGAAATCGC	60 °C
3	Synaptophysin (*Syn*) F primer *Syn* R primer	CATTCAGGCTGCACCAAGTG TGGTAGTGCCCCCTTTAACG	60 °C
4	Polysynaptic Density Protein (*PSD*) F primer *PSD* R primer	GGACATTCAGGCGCACAAG TCCCGTAGAGGTGGCTGTTG	60 °C
5	*GAPDH* F primer *GAPDH* R primer	ACCCAGAAGACTGTGGATGG CACATTGGGGTAGGAACAC	60 °C
6	n-Acetylcholinesterase α7 (*nAChR*) F primer α7 *nAChR* R primer	TGCAAAGAGCCATACCCAGA TGATCCTGGTCCACTTAGGC	60 °C
7	α4 *nAChR* F primer α4 *nAChR* R primer	GTCTAGAGCCCGTTCTGTGA TAGTCATGCCACTCCTGCTT	60 °C
8	β2 nAChR F primer β2 nAChR R primer	GATGACCAGAGTGTGAGGGA CCCCCACCGTTAACACTACT	60 °C
9	Choline Acetyltransferase (*ChAT*) F primer *ChAT* R primer	GGCTTACTACAGGCTTTACC CTGGTGGAGAGAATAAACCG	60 °C

## Data Availability

All the data related to this study are contained within this paper and nowhere else.

## Supplementary Materials

The following supporting information can be downloaded at https://www.mdpi.com/article/10.3390/biomedicines14102208/s1, Table S1: List of differentiated metabolites among Control, Met + HFD, Met + HFD + SEGE (2 mg/kg/day) and Met + HFD + SEGE (12 mg/kg/day) groups at *p* < 0.05 and Fold Change > 1.5. FC= Fold Change.

## Abbreviations

The following abbreviations are used in this manuscript:

Aβ	Amyloid Beta
ACh	Acetylcholine
AChE	Acetylcholinesterase
Al	Aluminum
ANOVA	Analysis of Variance
As	Arsenic
ASAB	Atta-ur-Rahman School of Applied Biosciences
BBB	Blood Brain Barrier
BDNF	Brain Derived Neurotropic Factor
CAMK-4	Calcium/Calmodulin-Dependent Protein Kinase-IV
CREB	Cyclic Adenosine Monophosphate Response Element Binding Protein
Cd	Cadmium
ChAT	Choline Acetyl Transferase
Cr	Chromium
Cu	Copper
DNA	Deoxyribonucleic Acid
EPA	Environmental Protection Agency
GAPDH	Glyceraldehyde-3-phosphate Dehydrogenase
GC/MS	Gas Chromatography Mass Spectrometry
GDP	Gross Domestic Production
GN	Gingerol
HEC	Higher Education Commission
HFD	High-Fat Diet
Hg	Mercury
HCL	Hydrochloric Acid
IRB	Institutional Review Board
Mn	Manganese
nAChR	Nicotinic Acetylcholine Receptors
NIH	National Institutes of Health
NRPU	National Research Program for Universities
NUST	National University of Sciences and Technology
Pb	Lead
PSD95	Polysynaptic Density Protein 95
qRT-PCR	Quantitative Reverse Transcription Real Time Polymerase Chain Reaction
RNA	Ribonucleic Acid
ROS	Reactive Oxygen Species
SEGE	Shogaol-Enriched Ginger Extract
SG	Shogaol
WHO	World Health Organization
