# Supplementary material for: The Neuroprotective Properties of Shogaol-Enriched Ginger Extract (SEGE) Mitigate Synaptic, Cholinergic, and Metabolic Brain Dysfunction in a Mouse Model of Metals and High-Fat-Diet-Induced Neuropathology
Source: Biomedicines. 2026 Sep 29;14(10):2208. doi: 10.3390/biomedicines14102208 (PMC13701904; doi:10.3390/biomedicines14102208)
Supplement: Supplementary file 1 [file biomedicines-14-02208-s001.zip › biomedicines-4373198-supplementary.pdf]

The Neuroprotective Properties of Shogaol-Enriched Ginger Extract (SEGE) Mitigate Synaptic, Cholinergic, and Metabolic Brain Dysfunction in a Mouse Model of Metals and High-Fat-Diet-Induced Neuropathology

Sara Ishaq [1,2], Armeen Hameed [1], Amna Liaqat [1], Rabia Basri [1], Sohana Siyar [1], Syed Ghulam Musharraf[3], Amna Jabbar Siddiqui[4], Zaman Ashraf [5,6], Sher Qadar [6], Touqeer Ahmed [1,*]

[1]Neurobiology Laboratory, Department of Biomedicine, Atta-ur-Rahman School of Applied Biosciences, National University of Sciences and Technology, Sector H-12, Islamabad 44000, Pakistan

[2]Planning Commission, Ministry of Planning, Development, & Special Initiatives (MoPD&SI), Government of Pakistan, Pak Secretariat, Islamabad 44000, Pakistan

[3]H.E.J. Research Institute of Chemistry, International Center for Chemical and Biological Sciences, University of Karachi, Karachi 75270, Pakistan

[4]Dr. Panjwani Center for Molecular Medicine and Drug Research, International Center for Chemical and Biological Sciences, University of Karachi, Karachi 75270, Pakistan

[5]Department of Chemistry, Faculty of Sciences, Rawalpindi Women University, Rawalpindi-46300, Pakistan

[6]Department of Chemistry, Allama Iqbal Open University, Islamabad 44000, Pakistan

*Correspondence: touqeer.aahmed@gmail.com; Tel.: +92-51-9085-6166

Supplementary Material:

Differentially Expressed & Identified Metabolites:

All the differentially expressed and identified metabolites are given in supplementary table S1 with their International Union of Pure and Applied Chemistry (IUPAC) International Chemical Identifier (InChI).

Table S1. List of Differentiated Metabolites among Control, Met+HFD, Met+HFD+SEGE (2mg/kg/day) & Met+HFD+SEGE (12mg/kg/day) Groups at $p < 0.05$ and Fold Change>1.5. FC= Fold Change.

**Sr. No.**	**(a) Compound or (b) Base peak (*m/z*)**	**Pathway & Function**	**Retention Time (min)**	**p (Corr)**	**Log FC (Met+HFD vs Control)**	**Log FC (Met+HFD+SEGE 2mg/kg/day vs Control)**	**Log FC (Met+HFD+SEGE 12mg/kg/day vs Control)**	**Upregulation/Downregulation compared to control group**		
								**Met+HFD**	**Met+HFD+SEGE (2mg/kg/day)**	**Met+HFD+SEGE (12mg/kg/day)**
**1**	1-(1-Ethylpropyl)-4-propylbenzene	Secondary metabolite in neurotransmitters breakdown	15.05	0	1.9282	0	1.3083	up	down	up
**2**	1,2-Benzenedicarboxylic acid, bis(2-ethylhexyl) ester	Neurotoxicant, activates chemical carcinogenesis pathway	35.03	0	1.9266	0	1.9028	up	down	up
**3**	10-Methyl-1-hexadecanol	Fatty acid and lipid metabolism product	19.41	0	2.2111	0	0	up	down	down

**4**	1-Methoxy-9-octadecene	Cell membrane structure and cell signaling pathway	27.86	0	0	0.6788	2.0292	down	up	up
**5**	1-Methoxymethyl-2,5-dimethylimidazole	Neurotransmitter synthesis and cell signaling pathway	7.243333	0	0	0	2.2109	down	down	up
**6**	2,3,6-Trimethyloctane	Lipid metabolism and energy storage	11.59667	0	2.2111	0	0	up	down	down
**7**	2,4-Lutidine	Aromatic compound involved in neurotransmitter formation and cellular signaling	7.018	0	-0.6599	-1.9483	-1.9483	down	down	down
**8**	2-Methyl-4-keto-pentane-2-ol	Secondary metabolite in various	8.257999	0	0.6141	-1.3076	-1.3076	up	down	down

		metabolic pathways and source of energy production during fasting								
**9**	3,4-Diethyl-2-methylpyrrole	Biosynthesis of secondary metabolites especially of neurotransmissio n	8.08	0	0	0	2.2103	down	down	up
**10**	3,4-Lutidine	Aromatic compound involved in in neurotransmitter formation and cellular signaling	7.033333	0	0	0	2.211	down	down	up
**11**	3,8-Dimethylundecane	Lipid metabolism and	18.68333	0	1.9484	0	1.8799	up	down	up

		cell membrane structures								
**12**	4,6-Dimethyldodecane	Lipid metabolism and cell membrane structures	13.3725	0	2.0323	0	0.6756	up	down	up
**13**	4,7-Dimethylundecane	Lipid metabolism and cell membrane structures	13.97667	0	2.0386	0.6693	0	up	up	down
**14**	4-Ethyl-1-octyn-3-ol	Enzymatic role in cellular metabolism and sometimes NMDA receptors antagonism	34.03333	0	0	0	2.2108	down	down	up
**15**	4-Methyl-1,2,4-triazole	Affect the enzymatic activity of different	6.455	1.69E-04	-2.4479	-1.9743	-1.6005	down	down	down

		enzymes and act as ligand for various brain receptors								
**16**	4-Pentadecyne, 15-chloro-	Chlorinated compound disturbs cellular functions	30.29	0	2.211	0	0	up	down	down
**17**	5-Methyl-4,5-methylenenonane	Secondary metabolite in various metabolic pathways of vitamins	26.93667	0	0	0	2.2111	down	down	up
**18**	6-ethyl-1,3-dimethylindan-5-carbaldehyde	Biosynthesis of secondary metabolites	18.22	0	0	0	2.2111	down	down	up
**19**	9,12-tetradecadienyl acetate	Secondary metabolite in TCA, also in	27	0	0	0	2.211	down	down	up

		cholinergic synapse, and hormonal receptors								
**20**	9-Tricosene	Lipid metabolism and cellular structures	35.37333	0	1.9147	0	1.9118	up	down	up
**21**	Alanine	Protein synthesis and neurotransmitter precursor	11.20083	0.00975	1.0717	2.3365	1.3271	up	up	up
**22**	Arabinofuranose	Amino sugar and nucleotide sugar metabolism	27.395	0	-2.051	-2.051	-0.2953	down	down	down
**23**	Benzene, 1-(5,5-dimethyl-1-cyclopenten-1-yl)-2-methoxy-	Product of chemical carcinogenesis and aromatic	18.125	0	0.6728	0	2.0351	up	down	up

		compounds degradation								
**24**	Benzoic acid, 4-ethoxy-, ethyl ester	Biosynthesis pathway of local analgesics and alkaloids	15.44083	0.01948	-0.9561	-0.5028	1.1774	down	down	up
**25**	Cholestene	Related to cholesterol metabolism	36.78	0	0	0.6676	2.0402	down	up	up
**26**	Cyclohexasiloxane, dodecamethyl-	Carrier for other compounds	12.5	0	2.0503	0	0.6572	up	down	up
**27**	Cyclopentasiloxane, decamethyl-	Crrier for other compounds	9.933333	0	1.9487	0	1.8797	up	down	up
**28**	Cyclotetrasiloxane, octamethyl-	Carrier for other compounds	9.85	0	0	0	2.2111	down	down	up
**29**	Dichloroacetic acid, 2-octyl ester	Cellular metabolic intermediate of chlorinated	15.84	0	1.9258	0	1.9038	up	down	up

		compounds and xenobiotics								
**30**	Dihydrophytol	Basically a phytol precursor in plants but also involved in antioxidant activities	19.05143	0	1.9185	0.644	1.9668	up	up	up
**31**	Di-isodecyl phthalate	Degradation product of aromatic compounds	26.24	0	0	0	2.2111	down	down	up
**32**	DODECANAL DIMETHYLACETAL	Aldehyde involved in metabolic process and signal transduction	30.59	0	1.9242	0.6609	1.9457	up	up	up
**33**	Dodecanamide	Involved in signal	33.38667	0	0	0	2.211	down	down	up

		transduction and serve as building blocks for larger molecules								
**34**	Eicosane	Long chain hydrocarbon involved in energy storage, lipid metabolism and cellular structures	21.468	0	1.2835	0	1.953	up	down	up
**35**	Ethyl 1-Hydroxy-3-(t-butyldimethylsilyl)cyclohex-4-en-2-carbamate	Interacts with cellular various enzymes and disturbs their function	7.916667	0	2.211	0	0	up	down	down
**36**	Glycine	Amino acid also work as an inhibitory	10.68	0	2.2103	0	0	up	down	down

		neurotransmitter in brain								
**37**	Hexacosane	Long chain hydrocarbon involved in energy storage and cellular structures	13.64	0	2.2111	0	0	up	down	down
**38**	Inositol	PI3K/Akt, Ras, phospholipase D, cholinergic synapse and many other signaling pathways	27.64333	0	1.46	-0.575	1.3305	up	down	up
**39**	Lactam	Antibiotic breakdown product, can also be involved in	8.843333	0	1.8136	0	2.0061	up	down	up

		signaling pathways								
**40**	Methyl behenate	Energy storage and cellular signaling, also high in cellular infections	34.75	2.10E-04	0.808	0.513	2.4439	up	up	up
**41**	Methyl erucate	Energy storage and cellular signaling	29.008	0	1.2761	0	1.9602	up	down	up
**42**	Methyl Heptadecanoate	Energy storage and cellular signaling	27.82	0	2.2105	0	0	up	down	down
**43**	Methyl isostearate	Energy storage and cellular signaling	29.83571	0	0.643	1.9235	1.9644	up	up	up
**44**	Methyl linoleate	Energy storage and cellular signaling	29.3	0	1.9142	0	1.9151	up	down	up

**45**	Methyl palmitate	Energy storage and cellular signaling	24.59333	1.06E-05	-1.6559	-1.4098	0.5875	down	down	up
**46**	Methyl stearate	Energy storage and cellular signaling	32.602	0	-2.0858	-2.0858	-0.9636	down	down	down
**47**	Myoinositol	Secondary metabolite in various cell signaling pathways	27.64333	0	-2.2111	-2.2111	-2.2111	down	down	down
**48**	N-Methyl-N-butyltrifluoroacetamide	Cellular metabolism	10.735	0	1.9247	0	1.9016	up	down	up
**49**	Nonadecane	Energy storage, lipid metabolism, and cellular structures	20.268	0	1.9495	0	1.2818	up	down	up

**50**	Oleic acid	Energy metabolism and cellular membrane structures	30.82	0	0	1.9977	1.2351	down	up	up
**51**	Oxalic acid, 2-phenylethyl tridecyl ester	Building blocks of large cellular molecules, also involved in cellular metabolism	13.75	0	2.2111	0	0	up	down	down
**52**	Palmitic acid	Energy metabolism and cell membrane structures	31.71	0	1.3312	-0.6151	1.3523	up	down	up
**53**	Palmitic acid ethyl ester	Energy metabolism and cell membrane structures	31.71	0	0	2.211	0	down	up	down

**54**	Palmitic acid vinyl ester	Energy metabolism and cell membrane structures	31.71	0	0	0	2.2111	down	down	up
**55**	Pelargol	Unknown	8.986667	0	1.9202	0	1.9094	up	down	up
**56**	Pentadecanal	Metabolic processes and signal transduction	20.93572	0	1.9186	0.6765	1.9343	up	up	up
**57**	Phytan	Glycerophospholipid metabolism and secondary metabolites metabolism	15.785	0	0.6798	0	2.0277	up	down	up
**58**	PYRIDINE	Involved in the biosynthesis of various neurotransmitters	6	0	2.2111	0	0	up	down	down

		and vitamins metabolism								
**59**	Sulfurous acid, 2-propyl tridecyl ester	Secondary metabolite	29.856	0	1.2788	0	1.9577	up	down	up
**60**	Sulfurous acid, butyl heptadecyl ester	Secondary metabolite	32.53714	0	1.9705	0.6497	1.9099	up	up	up
**61**	Tetracosane	Energy metabolism and cellular structures	27.96833	0	1.938	0	1.8889	up	down	up
**62**	Tetradecane	Energy metabolism and cellular structures	13.9525	0	0.7122	0	1.993	up	down	up
**63**	Triacetonamin	Decrease cellular malonaldehyde	10.268	0	0.4786	-1.375	-1.375	up	down	down
**64**	Tricosane	Energy metabolism and	19.16834	0	1.8873	0	1.9412	up	down	up

		cellular structures								
**65**	Tridecane	Energy metabolism and cellular structures	15.786	0	1.9407	0	1.296	up	down	up
**66**	Trimethyl - benzene	Cell signaling	7.36	0	2.0205	0	0.6873	up	down	up
**67**	Tritriacontane	Energy metabolism and cellular structures	31.20286	0	1.8825	0.6641	1.9806	up	up	up
**68**	Undecane	Energy metabolism and cellular structures	13.48333	0	0	0	2.2103	down	down	up
**69**	208.0@39.1625	Not known	39.1625	0	2.0089	0.6975	0	up	up	down
**70**	56.0@11.04	Not known	11.04	0	2.211	0	0	up	down	down
**71**	57.0@37.82	Not known	37.82	0	0.652	0	2.0548	up	down	up

**72**	81.0@39.69	Not known	39.69	0	2.211	0	0	up	down	down
